# The origin and biosynthesis of the naphthalenoid moiety of juglone in black walnut

**DOI:** 10.1038/s41438-018-0067-5

**Published:** 2018-11-01

**Authors:** Rachel M. McCoy, Sagar M. Utturkar, Joseph W. Crook, Jyothi Thimmapuram, Joshua R. Widhalm

**Affiliations:** 1 Department of Horticulture and Landscape Architecture, Purdue University, 625 Agriculture Mall Drive, West Lafayette, IN 47907 USA; 2 Purdue Center for Plant Biology, Purdue University, West Lafayette, IN 47907 USA; 3 Bioinformatics Core, Purdue University, 155 South Grant Street, West Lafayette, IN 47907 USA

## Abstract

Several members of the *Juglandaceae* family produce juglone, a specialized 1,4-naphthoquinone (1,4-NQ) natural product that is responsible for the notorious allelopathic effects of black walnut (*Juglans nigra*). Despite its documented ecological roles and potential for being developed as a novel natural product-based herbicide, none of the genes involved in synthesizing juglone have been identified. Based on classical labeling studies, we hypothesized that biosynthesis of juglone’s naphthalenoid moiety is shared with biochemical steps of the phylloquinone pathway. Here, using comparative transcriptomics in combination with targeted metabolic profiling of 1,4-NQs in various black walnut organs, we provide evidence that phylloquinone pathway genes involved in 1,4-dihydroxynaphthoic acid (DHNA) formation are expressed in roots for synthesis of a compound other than phylloquinone. Feeding experiments using axenic black walnut root cultures revealed that stable isotopically labeled l-glutamate incorporates into juglone resulting in the same mass shift as that expected for labeling of the quinone ring in phylloquinone. Taken together, these results indicate that *in planta*, an intermediate from the phylloquinone pathway provides the naphthalenoid moiety of juglone. Moreover, this work shows that juglone can be *de novo* synthesized in roots without the contribution of immediate precursors translocated from aerial tissues. The present study illuminates all genes involved in synthesizing the juglone naphthoquinone ring and provides RNA-sequencing datasets that can be used with functional screening studies to elucidate the remaining juglone pathway genes. Translation of the generated knowledge is expected to inform future metabolic engineering strategies for harnessing juglone as a novel natural product-based herbicide.

## Bioengineering: revealing the genetic roots of a potential natural herbicide

Revealing the synthetic pathway of the natural herbicide juglone paves the way for genetically engineered, weed-resistant crops. Juglone, belonging to the same molecular family as phylloquinone (also known as vitamin K_1_), is produced by plants, including black walnut and is generally toxic to nearby plant life. Until now, the genetic and synthetic basis of juglone remained unknown. Using genetic analysis, a research team from the United States’ Purdue University, led by Joshua Widhalm, discovered that juglone is actually a product of the same synthetic pathway as phylloquinone and shares the same progenitor—a molecule called DHNA. This study partially reveals the genes responsible for juglone synthesis, and the authors suggest that the next step involves elucidating how the chemical is produced from DHNA. This discovery informs future efforts to engineer resilience into important crops.

## Introduction

**Fig. 1:**
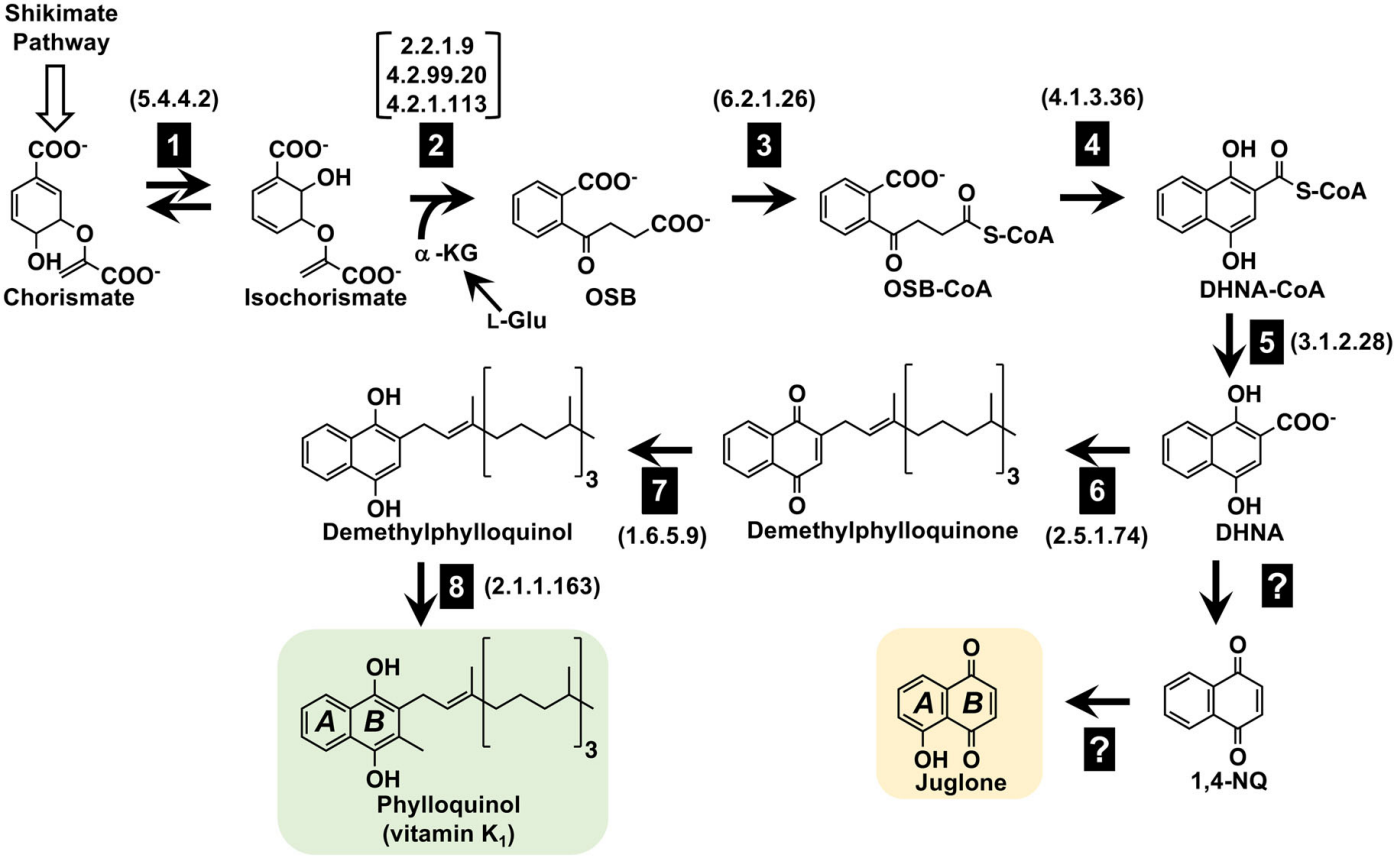
The proposed connection between phylloquinone and juglone biosynthesis in *Juglans nigra* (black walnut). Presented is the phylloquinone pathway as elucidated via genetic and biochemical studies in *Arabidopsis thaliana*. The product of the phylloquinone pathway is its reduced form, phylloquinol, which spontaneously re-oxidizes to phylloquinone in the presence of molecular oxygen. We hypothesize that in black walnut, the naphthalenoid moiety of juglone originates from 1,4-dihydroxynaphthoic acid (DHNA) derived from the phylloquinone pathway. Subcellular architecture is not depicted. Boxed numbers next to arrows indicate identified enzymes: 1, isochorismate synthase; 2, PHYLLO, trifunctional 2-succinyl-5-enolpyruvyl-6-hydroxy-3-cyclohexene-2-carboxylate (SEPHCHC) synthase, 2-succinyl-6-hydroxy-2,4-cyclohexadiene-2-carboxylate (SHCHC) synthase, and *o*-succinylbenzoate (OSB) synthase; 3, OSB-CoA ligase; 4, 1,4-dihydroxy-2-naphthoyl-CoA (DHNA-CoA) synthase; 5, DHNA-CoA thioesterase; 6, DHNA phytyl transferase; 7, NAD(P)H dehydrogenase C1 (NDC1); 8, demethylphylloquinone methyltransferase. Numbers in brackets next to arrows indicate official Enzyme Commission numbers. Open block arrow indicates steps of the shikimate pathway. α-KG α-ketoglutarate, 1,4-NQ 1,4-naphthoquinone, l-Glu L-glutamate

The allelopathic effects of black walnut (*Juglans nigra*) on numerous types of plants growing within the span of its canopy and root system have been reported since antiquity^1^ and remain a concern of backyard growers today^2^. It is now established that the natural product juglone (5-hydroxy-1,4-naphthoquinone; Fig. 1), released into the soil through decaying litterfall, root contact, or rain leaching from its drip line, is responsible for the detrimental effects elicited by black walnut on other species. Juglone is also produced in significantly lower quantities by several other members of the Juglandaceae family, including English or Persian walnut (*J. regia*), Japanese walnut (*J. sieboldiana*), butternut (*J. cinerea*), pecan (*Carya illinoensis*), and hickory (*Carya ovata*)^3^. Classically, the effects of juglone toxicity on susceptible species have been characterized as leaf wilting and yellowing eventually resulting in death^4^, but recently juglone was also found to damage roots through induction of reactive oxygen and nitrogen species, as well as through calcium accumulation^5,6^. Though many plants are susceptible to juglone, some commonly grown garden vegetables (e.g., squash), fruits (e.g., cherry), landscape plants (e.g., crabapple), flowers (e.g., tulip), and grasses (e.g., Kentucky bluegrass) are reportedly tolerant^2^. Together with the fact that juglone inhibits the growth of several common weed species^7^, this raises the prospect of developing juglone as a novel natural product-based herbicide. However, the absence of knowledge about the genes involved in synthesizing juglone precludes designing strategies to metabolically engineer juglone production in economically important crops or in large-scale biological platforms.

Juglone belongs to a class of redox active compounds called the 1,4-naphthoquinones (1,4-NQs). The 1,4-NQs structurally consist of a benzene ring that is linearly fused with a fully conjugated cyclic diketone bearing carbonyl groups arranged in the *para* orientation^8^. All plants synthesize phylloquinone (vitamin K_1_; Fig. 1), a methylated and phytyl-conjugated 1,4-NQ serving as a one-electron carrier at the A1 site of photosystem I (PSI)^9^. The phylloquinone pathway (Fig. 1) consists of 10 enzymatic reactions starting from chorismate, the final product of the shikimate pathway^10^. Chorismate is isomerized to isochorismate by isochorismate synthase (ICS), an enzyme shared with salicylic acid biosynthesis^11,12^. Next, α-ketoglutarate is decarboxylated to form a succinic semialdehyde, which is condensed with isochorismate and the resulting intermediate is converted to *o*-succinylbenzoic acid (OSB) by a trifunctional enzyme called PHYLLO^11^. The succinyl side chain of OSB is then activated by an OSB-CoA ligase^13^ and cyclized by naphthoate synthase to form 1,4-dihydroxy-2-naphthoyl-CoA (DHNA-CoA), which is hydrolyzed to 1,4-dihydroxynaphthoic acid (DHNA) by DHNA-CoA thioesterase^14^. Through a sequential series of phytylation^15^, reduction^16^, and methylation^17^ reactions, DHNA is finally converted to phylloquinone (Fig. 1)^18^.

Early radiotracer studies using English walnut leaves revealed that the benzene ring (ring *A*, Fig. 1) of juglone is derived from shikimate^19^. Labeling experiments later showed that OSB^20^ and DHNA^21^ can be incorporated into juglone, suggesting that juglone’s quinone ring (ring *B*, Fig. 1) originates from l-glutamate via α-ketoglutarate and leading us to hypothesize that juglone biosynthesis branches off the phylloquinone pathway (Fig. 1). To investigate if the juglone and phylloquinone pathways share common biosynthetic genes to synthesize their naphthalenoid moieties, we used targeted metabolic profiling and comparative RNA sequencing (RNA-seq) to examine the co-occurrence between 1,4-NQ natural product pools and expression of phylloquinone pathway genes in organs of black walnut, the species producing the highest known levels of juglone^3^. We then tested if stable isotopically labeled glutamate fed to axenic black walnut root cultures is incorporated into juglone with the same mass shift as that expected if juglone is derived from an intermediate of the phylloquinone pathway. The obtained results and generated transcriptomes are expected to serve as useful resources for future studies aimed at elucidating the remainder of the juglone pathway and for uncovering genes involved in its transport and sequestration. These advances in basic knowledge will inform the development of biotechnological approaches for harnessing juglone as a novel natural product-based herbicide.

## Materials and methods

### Plant materials and general experimental procedures

Tissues used in this study were collected from 1-year-old (leaves, stems, bark, and roots) and mature elite (flowers and hulls) black walnut trees located at Martell Forest (West Lafayette, IN, USA). Naphthoquinone standards of juglone, phylloquinone, menaquinone-4 (MK-4), and plumbagin were from Sigma-Aldrich. Unless otherwise mentioned, all other reagents were from Fisher Scientific. Ultrapure water and high-performance liquid chromatographic (HPLC)- or gas chromatographic (GC)-grade solvents were used for all metabolite extractions. HPLC analyses were carried out on an Agilent 1260 Infinity series instrument (Agilent Technologies) equipped with diode array and fluorometer detection modules employing Chemstation software. An Agilent 7890B GC coupled with a 5977A mass spectrometer (MS) employing Chemstation software was used to perform GC-MS analyses.

### Metabolic profiling

All steps were conducted in dimmed light to limit photodegradation of naphthoquinones. Approximately 500 mg of flash-frozen ground black walnut organs (young bark, female flowers, pollinated female flowers, male flowers, leaves, roots, and 1-year-old stems with the bark removed) were extracted in 10 mL methanol containing internal standards (1.8 nmol MK-4 and 200 nmol plumbagin) overnight with moderate shaking. To quantify juglone, 1 mL of the methanolic extract was filtered using a 0.2 μm polytetrafluoroethylene (PTFE) syringe filter and 10 μL directly analyzed by HPLC on a Zorbax SB-C18 column (4.6 × 250 mm, Agilent) thermostated at 40 °C and eluted in gradient mode at 0.75 mL min^−1^. The gradient started at 80% A (50 mM sodium acetate in water, pH 5.9) and 20% B (100% methanol) and linearly increased to 45% B over 4 min. From 4 to 17 min, the %B linearly increased from 45 to 80%. From 17 to 20 min, the %B linearly increased to 100%, and then returned to 20% B from 20 to 23 min. Finally, the column was washed in 20% B for 3 min. Juglone (15.7 min) and plumbagin (20.1 min) were detected fluorometrically (at 230 nm excitation and 372 nm emission) after reduction in a post-column chemical reactor (1.5 × 70 mm) packed with −100 mesh zinc (Sigma-Aldrich). Juglone and plumbagin were quantified relative to external calibration standards, and juglone content was corrected according to the recovery of the plumbagin internal standard.

To quantify phylloquinone, 3 mL of the methanolic extract was transferred to a pyrex tube, evaporated to near dryness under gaseous N_2_, resuspended in 2 mL 50% methanol, and partitioned twice with 3 mL hexanes. Upper phases from each partitioning were combined, transferred to new pyrex tubes, dried under gaseous N_2_, resuspended in 1 mL methanol, and 50 μL analyzed by HPLC-fluorescence as described previously^14^.

### RNA extraction, library construction, and sequencing

For RNA-seq experiments, RNA was extracted from roots and mature leaves of 1-year-old black walnut trees using the protocol described in Kolosova et al.^22^ except that partitioning was done with phenol/chloroform/isoamyl alcohol (25:24:1 v/v/v). Samples were DNase-treated (NEB) according to the manufacturer’s instructions. A total of six cDNA libraries from three biological replicates, each replicate containing RNA pooled from two unique trees, of *J. nigra* roots and leaves were constructed using an mRNA-seq Kit (Illumina, San Diego, CA), and 101-bp paired-end reads were generated via Illumina HiSeq2500 at the Purdue Genomics Center, with at least 67 million reads per library. Sequence quality was assessed by FastQC (v. 0.10.0; http://www.bioinformatics.babraham.ac.uk). The raw data were submitted to the Sequence Read Archive (http://www.ncbi.nlm.nih.gov/sra/) and are available under the accession number SRP133522.

### Bioinformatics analysis

Quality-based trimming of raw sequence data was performed using FASTX toolkit (version 0.0.14; http://hannonlab.cshl.edu/fastx_toolkit/) and reads ≥ 50 bp and bases with quality value ≥ 30 were retained. Reads mapping to complete chloroplast or mitochondrial genomes of closely related species *J. regia* and *Liriodendron tulipifera* were removed. The overall mapping rate of the six replicates against the *J. regia* genome^23^ was in the range of 57–64%, while the rate against transcriptome from *de novo* assembly of clean reads performed using the Trinity^24^ (version 2.2.0) transcriptome assembler was 76–85%. Therefore, to include more reads in the analysis, the *de novo* assembly results were used. Redundant transcripts were removed by clustering through CD-HIT tool^25^ (version 4.6.5) with default parameter and at least 90% alignment coverage. After clustering, transcripts shorter than 500 bp were excluded from the assembly. These non-redundant transcripts were used for annotation using blastx against the NCBI-NR, *Arabidopsis thaliana* and *J. regia* protein databases.

Clean reads were mapped back to the non-redundant transcripts from the *de novo* assembly and transcript abundance at gene level was estimated using the RSEM package (version 1.2.30)^26^ with default parameters. RSEM determines a normalized measure of transcript expression and estimates read counts associated with each gene feature. Counts matrix generated by RSEM was used as input for EBSeq^27^ and DESeq2^28^ methods to determine differentially expressed genes (DEGs). A false discovery rate (FDR) of 0.05 was selected as cutoff to identify significantly DEGs.

Gene Ontology (GO) and Kyoto Encyclopedia of Genes and Genomes (KEGG) terms associated with each gene were determined from the *J. regia* and Arabidopsis blast hit descriptions, respectively. GO and KEGG enrichment analysis was performed using the Bioconductor package clusterProfiler^29^ and terms with adjusted *p*-values of ≤0.05 were defined as significantly enriched.

### Quantitative PCR analysis

Quantitative PCR (qPCR) was used to validate the expression patterns found for the phylloquinone genes in black walnut. Primers were designed using PrimerExpress (ThermoFisher). qPCR reactions were performed using a StepOnePlus (ThermoFisher) in a 20 μL reaction as follows: 10 μL of 5× Fast SYBR Green PCR master mix (ThermoFisher); 2 μL each of the forward and reverse primers (50–900 nM final concentration, Supplementary Table S1); 4 μL of cDNA diluted from RNA-seq libraries; and 2 μL of water. Expression was normalized to the *J. nigra* ubiquitin carrier protein.

### Stable isotope labeling of juglone by feeding ^13^C-glutamate

Young, non-woody roots collected from 1-year-old black walnut trees were excised, rinsed, and cut into 1 cm sections. Roots were sterilized by pre-treatment in 10 mL Murashige and Skoog (MS) containing 5% (v/v) PPM^TM^ (Plant Cell Technology) for 1 h. Approximately 150–250 mg of sterilized roots were transferred into 10 mL of half-strength MS media (pH 5.7) containing 3% sucrose, 0.1% PPM, and 25 mM glutamate-[^13^C_5_^15^N_1_] (Cambridge Isotopes). Roots were incubated in darkness at 28 °C with moderate shaking (80 rpm). Root tissue and media were separately collected after 1, 6, 12, 24, 48, and 96 h and flash-frozen until analysis by HPLC coupled with fluorescence detection (HPLC-FLD) and GC-MS. Fed roots were extracted with 10 mL methanol, spiked with 125 nmol α-aminoadipate and 200 nmol plumbagin internal standards, and incubated overnight at 4 °C in the dark. To measure juglone pool sizes, 500 μL of the methanolic extract was filtered using a 0.2 μm PTFE syringe filter and 10 μL was analyzed by HPLC-fluorescence as described above. Isotopic abundance of juglone was measured by directly analyzing 1 μL by GC-MS and an Agilent 19091s-433 HP-5MS capillary column (30 m × 0.25 mm; film thickness 0.25 μm). A volume of 1 µL was injected in split mode with a 1:50 split ratio. Injector temperature was 260 °C. Column temperature was initially held at 35 °C for 3 min and then heated 8 °C min^−1^ to a final temperature of 260 °C, where it was held for 1 min. Helium was used as the carrier gas at a flow rate of 0.8 mL min^−1^. Electron ionization was set to 70 eV. Mass spectrum were obtained in scanning mode from 40 to 400 atomic mass units (amu). Juglone was identified by comparing retention time and mass spectra to an authentic standard.

To determine pool sizes and analyze isotopic abundances of glutamate the remaining 9.5 mL of the original methanolic extract was partitioned with 6 mL water and 5 mL of chloroform. After vortexing to form an emulsion, the mixture was incubated at least 1 h at 4 °C in the dark to allow phase separation to occur. The aqueous phase was transferred to a new vial and dried to completeness under air. Glutamate was extracted from the aqueous phase in a procedure modified from Rhodes et al.^30^. Briefly, the dried samples were resuspended in 1 mL of water and applied to Dowex-1-acetate 200 mesh column. Neutral and basic amino acids were washed off the column with 8 mL water. Acidic amino acids were eluted with 8 mL 0.2 M acetic acid and dried under air. Dried extracts were resuspended in 1 mL of water, applied to Dowex-50-H^+^ 200 mesh columns, and eluted with 6 mL of 6 M NH_4_OH. The eluant was then dried, resuspended in 400 μL 60% methanol, and dried again. To derivatize, samples were resuspended in 100 μL 5:1 isobutanol:acetyl chloride and heated at 120 °C for 20 min. Samples were dried, and 50 μL of heptafluorobutyric anhydride was added, followed by heating at 120 °C for 10 min. Samples were dried and resuspended in 100 μL 1:1 ethyl acetate:acetic anhydride. Samples were analyzed via GC-MS as previously described^30^ using α-aminoadipate as an internal standard.

To determine isotopic abundances, the percentage of juglone and glutamate labeling was calculated as the sum of the intensities of the shifted molecular ions divided by the sum of intensities for unshifted and shifted molecular ions, after correcting for natural isotope abundance. Labeled glutamate exhibited a shift of +6 amu, and to capture the interconversion between its amino and keto acid forms, +5 amu was also examined. Labeled juglone exhibited a shift of +3 amu.

## Results

### Spatial accumulation of juglone and phylloquinone in black walnut tissues

**Fig. 2:**
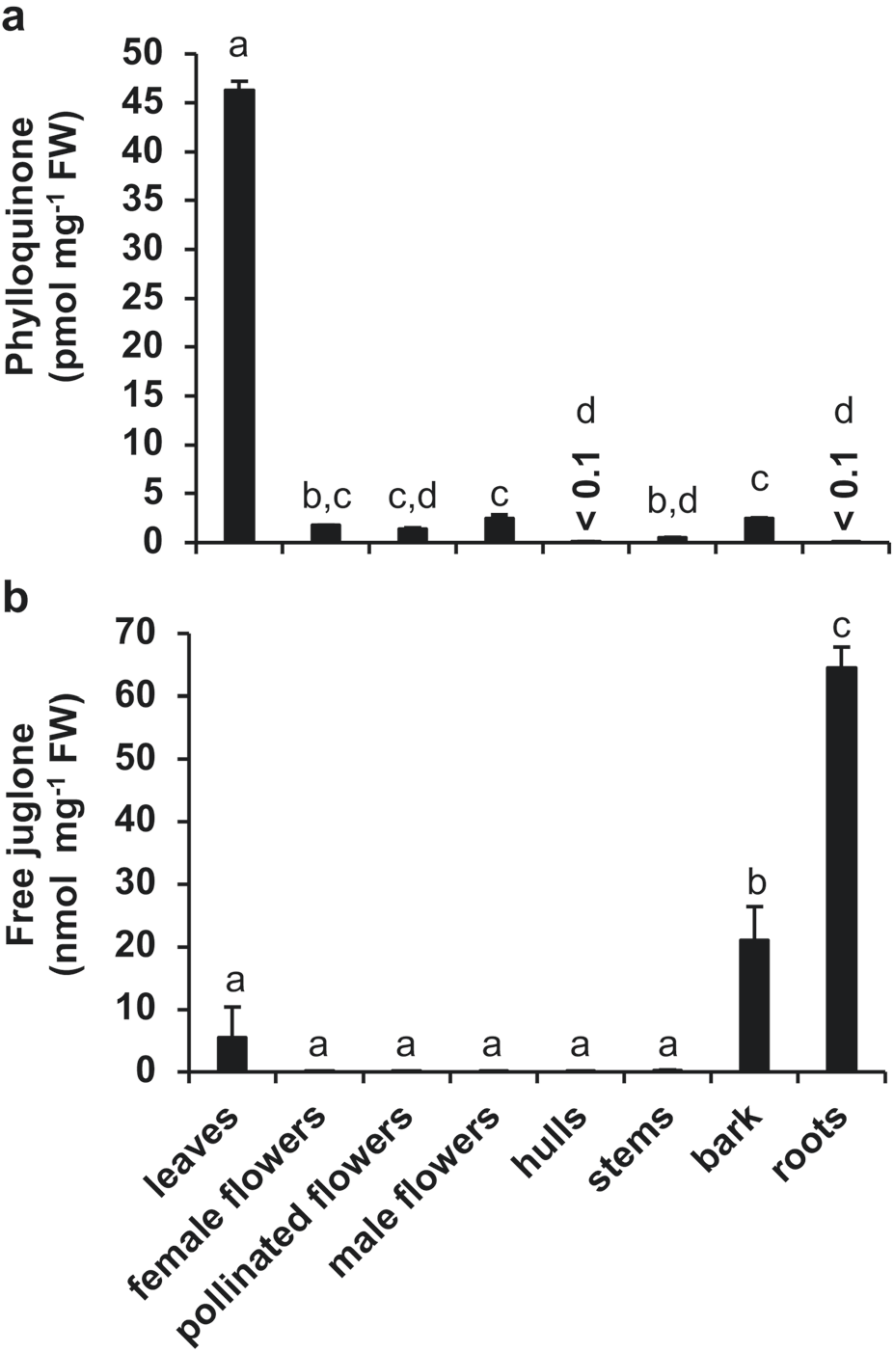
Pool sizes of phylloquinone and free juglone in *Juglans nigra* (black walnut) organs. **a** Phylloquinone levels. **b** Free juglone levels. All data are means ± SEM (*n* = ≥ 3 biological replicates). Different letters indicate significant differences via analysis of variance (ANOVA) followed by post-hoc Tukey test (*α* = 0.05)

When first considering our hypothesis that biosynthesis of the juglone naphthalenoid backbone branches from the phylloquinone pathway, we measured the spatial abundance of these two 1,4-NQ natural products in various black walnut tree tissues (see Materials and methods for tissue sources) using HPLC coupled with fluorescence detection. This method relies on the property of the 1,4-NQ ring to fluoresce when in its reduced form and allows for sensitive and selective detection without preparation of lipid-enriched fractions prior to analysis. Previously, it was shown that the reduction of phylloquinone needed for detection by HPLC-fluorescence can be achieved using an in-line post-column dry reactor packed with zinc dust^31^. We applied the same approach to achieve in-line conversion of juglone to its reduced form, hydrojuglone, during HPLC analysis, thereby allowing more sensitive and selective detection and quantification by fluorescence (Supplementary Figure S1). The pool size of phylloquinone was found to be highest in black walnut leaves (46.2 pmol mg^−1^ fresh weight, FW), below 3.5 pmol mg^−1^ FW in all flowers, hulls, peeled stems, and bark, and near the detection limit (<0.1 pmol mg^−1^ FW) in hulls and roots (Fig. 2a). At the same time, the free (metabolically active) juglone pool was determined to be highest in roots (64.5 nmol mg^−1^ FW), intermediate in bark (21.0 nmol mg^−1^ FW), and below 5.5 nmol mg^−1^ FW in all other tissues (Fig. 2b). These results indicate that the relative pool sizes of juglone and phylloquinone are inversely correlated to one another in roots and in leaves. Thus, these tissues were selected for generation of RNA-seq datasets to investigate the spatial expression pattern of phylloquinone pathway genes.

### Transcriptome sequencing, quality control, *de novo* assembly, and annotation

We generated RNA-seq datasets from *J. nigra* roots and leaves because they were found to be the tissues containing the lowest and highest levels of phylloquinone, respectively, and the highest and lowest levels of free juglone, respectively (Fig. 2 and Supplementary Figure S2). Six cDNA libraries prepared from RNA isolated from roots (RJn1, RJn2, and RJn3) and leaves (LJn1, LJn2, and LJn3) of 1-year-old *J. nigra* trees were subjected to Illumina 101-bp paired-end sequencing with over 436 million reads produced (Supplementary Table S2). After quality control, a total of 290 million clean reads for roots and 292 million clean reads for leaves remained and were assembled into 131 449 non-redundant transcripts (≥500 bp; N50 was 1819 bp). Out of 131 449 transcripts, 106 968 (81%) were found to have blastx hits against the NCBI non-redundant protein database, 105 376 (80%) against the *J. regia* protein database, 96 926 (73%) against the Arabidopsis protein database, and 89 338 (67%) against all three databases. These 131 449 transcripts correspond to 59 254 unique genes, which were used for all subsequent downstream analysis.

### Summary and GO enrichment analyses of DEGs

**Fig. 3:**
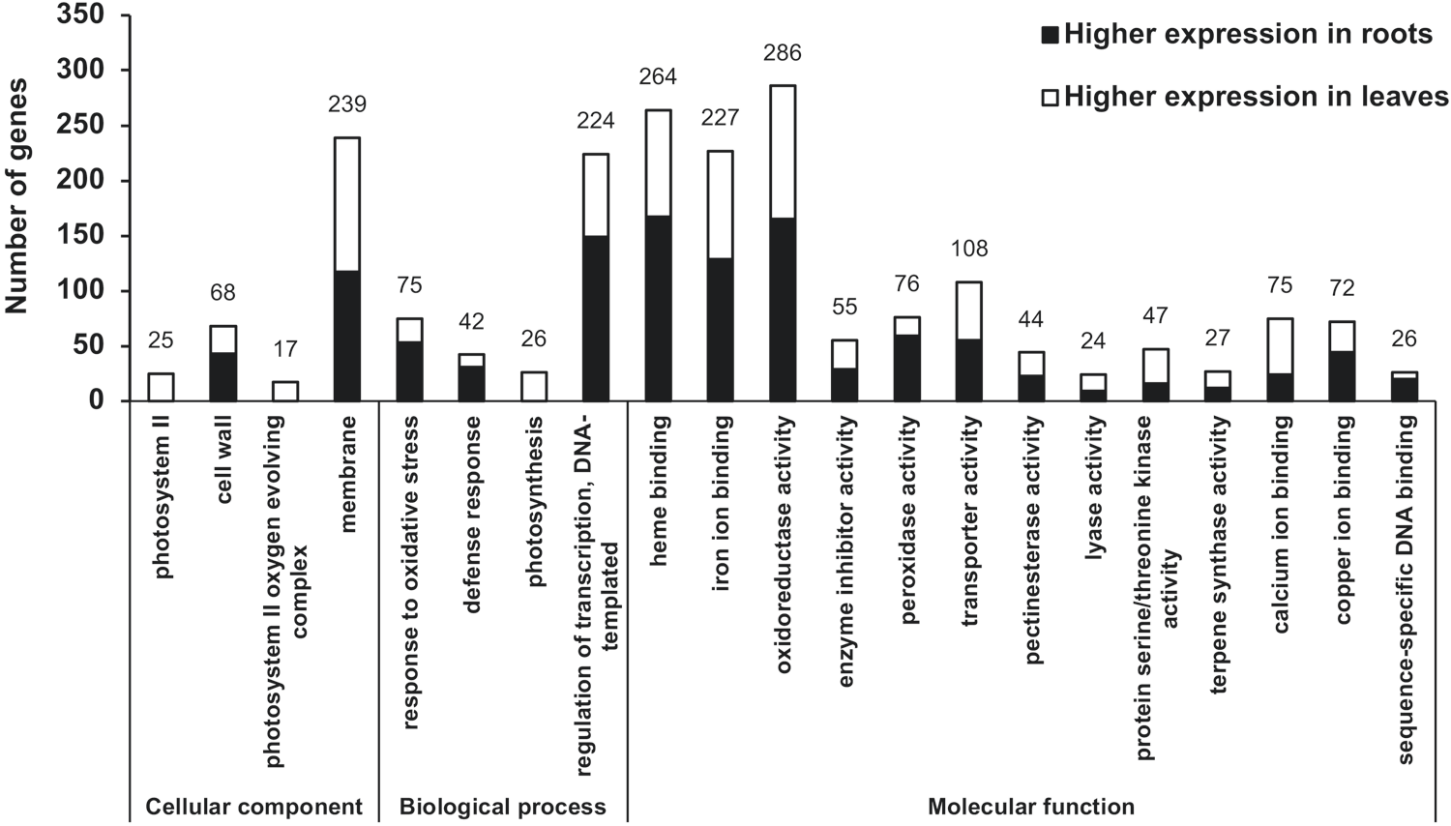
GO term enrichment in DEGs between roots and leaves of *Juglans nigra* (black walnut). Black shading in bars indicates higher expression in roots, and white shading indicates higher expression in leaves. Numbers above bars are total DEGs for each GO term

A total of 19 415 and 16 208 DEGs were detected in roots compared to leaves (FDR ≤ 0.05) using EBSeq and DESeq2, respectively. After combining the DEGs from both methods, a total of 21 608 unique DEGs were determined. We next used GO enrichment analysis to examine differences in the functional profiles of the root and leaf transcriptomes. GO terms were assigned to DEGs based on *J. regia* annotation and classified into Cellular Component, Biological Process, and Molecular Function groups (Fig. 3). In the Cellular Component group, 25, 17, 68, and 239 genes were assigned to the enriched categories photosystem II (PSII), PSII oxygen-evolving complex, cell wall, and membrane, respectively. Consistent with the absence of photosynthesis in roots, mRNA levels of all genes represented in the PSII and PSII oxygen-evolving complex categories were found to be reduced in roots compared to leaves with DESeq2 log_2_ fold-differences (log_2_FDs) ranging from 2.0 to 10.9 (Supplementary Tables S3 and S4). Similarly, in the Biological Process group, transcript levels of all 26 genes in the photosynthesis category were observed to be lower in roots compared to leaves with DESeq2 log_2_FDs ranging from 2.4 to 11.0 (Supplementary Tables S5 and S6). Also, in the Biological Process group, 75, 42, and 224 genes were assigned to the response to oxidative stress, defense response, and regulation of transcription categories, respectively (Fig. 3).

The most abundant GO terms assigned (FDR ≤ 0.05) to the Molecular Function group were heme binding, iron binding, and oxidoreductase activity, to which 264, 227, and 286 genes were associated, respectively. The mRNA levels for a majority of the genes in each of these categories were found to be higher in roots compared to leaves (Supplementary Tables S7 and S8). Moreover, many of the genes within the heme binding, iron binding, and oxidoreductase activity categories were assigned to all three categories and were annotated to encode pigment absorbing at 450 nm (P450) enzymes. P450s share a catalytic center comprised of an iron-coordinated heme, and to be active, P450s must also be coupled with electron-donating proteins, such as P450 reductases^32^. It is suggested that the hydroxylation of 1,4-NQ to form juglone (Fig. 1) could be catalyzed by a member of the P450 superfamily^8^. Therefore, P450 genes overrepresented in roots should be considered among candidates for the 1,4-NQ hydroxylase.

Several of the genes assigned to the heme binding category in the Molecular Function group were also assigned to the peroxidase activity category. Peroxidases are found in all plant organs but are particularly abundant in roots where they function in processes such as root hair growth and lignin formation^33^. Gene lists for the other significantly enriched categories in the Molecular Function group are provided in Supplementary Tables S7 and S8. Taken together, the GO enrichment analyses revealed typical functional profiles expected from root and leaf transcriptomes.

### Phylloquinone pathway gene expression analysis

**Fig. 4:**
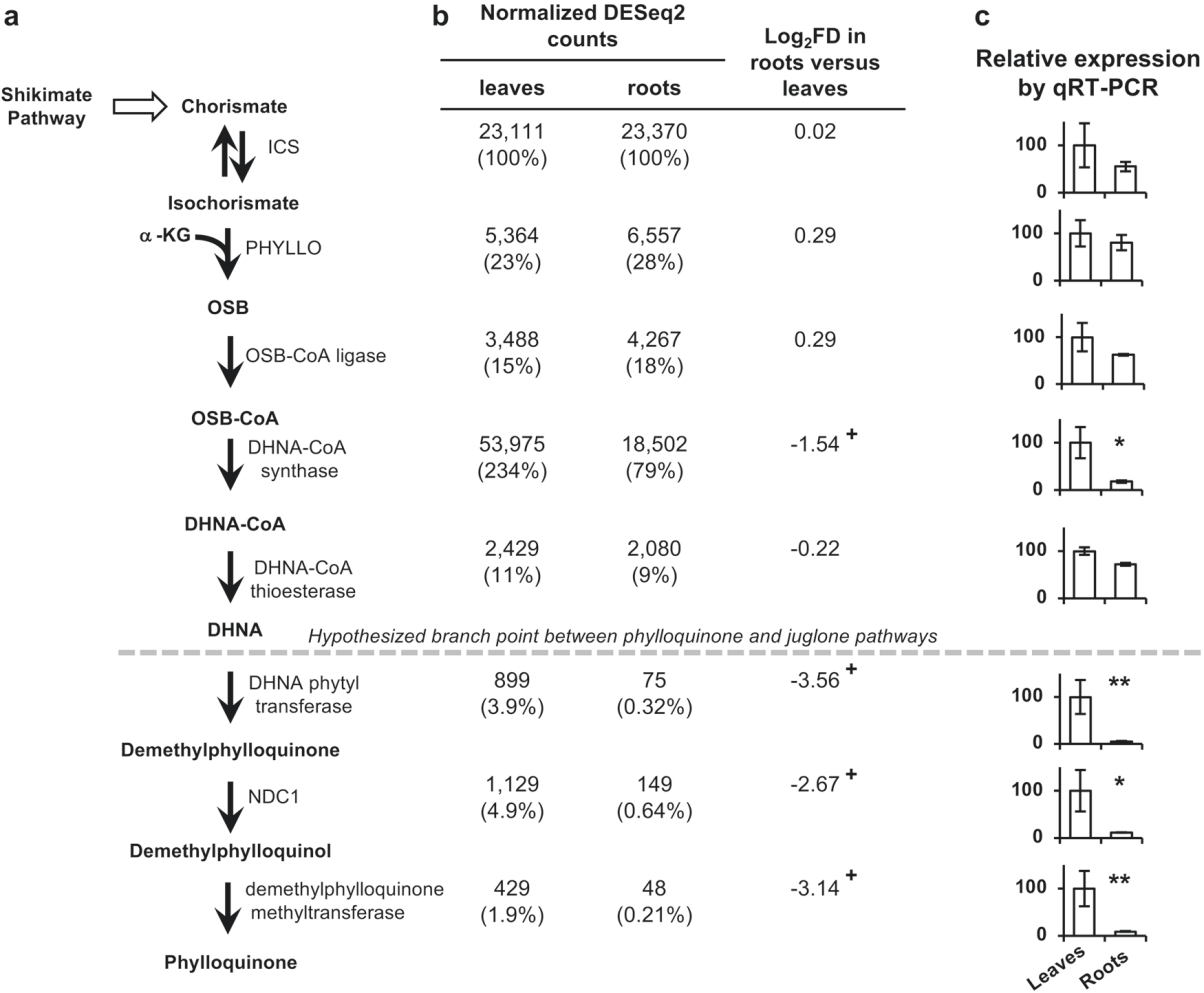
Relative expression pattern of phylloquinone pathway genes in *Juglans nigra* (black walnut) roots and leaves. **a** The phylloquinone pathway. See Fig. 1 legend for abbreviations. **b** Expression pattern of phylloquinone pathway genes based on normalized DESeq2 counts. Log_2_ fold-differences (FD) in roots compared to leaves are presented with negative numbers, which indicate lower mRNA accumulation in roots. Numbers in brackets are percent expression relative to ICS expression, based on normalized DESeq2 counts in the cognate organ. + indicates significant differential expression in leaves versus roots as calculated by DESeq2 (*p* < 0.001). **c** Relative gene expression in roots compared to leaves set at 100% as inferred by qPCR. Data are means ± SEM (*n* = 3 biological replicates), *p < 0.05 and **p < 0.01 by Student’s *t*-test relative to leaves

In Arabidopsis and *Zea mays* (maize), there are no known alternative fates of phylloquinone pathway intermediates downstream of isochorismate, which is precursor to certain hydroxybenzoic acids, including salicylic acid, and their cognate catabolites^34^. Accordingly, expression levels of phylloquinone pathway genes are generally much lower in roots compared to leaves in Arabidopsis and maize (Supplementary Figure S3). In black walnut roots, on the other hand, juglone is present at nanomolar levels compared to sub-picomolar levels of phylloquinone (Fig. 2 and Supplementary Figure S2). We therefore hypothesized that if juglone is *de novo* synthesized in roots using enzymes shared with the phylloquinone pathway, then the relative root versus leaf expression profile for genes involved in forming the naphthalenoid moiety of phylloquinone should be markedly higher in black walnut compared to Arabidopsis and maize. Using the amino-acid sequences of Arabidopsis and English walnut phylloquinone pathway enzymes as query, we first performed a tBlastn search to identify the phylloquinone pathway genes in our black walnut transcriptomes (Supplementary Table S9). Then, because RNA-seq is a quantitative approach, gene expression in roots relative to leaves was assessed based on the number of normalized counts corresponding to each gene in the generated datasets. This analysis revealed that expression levels of black walnut genes encoding ICS, the trifunctional enzyme PHYLLO, OSB-CoA ligase, and DHNA-CoA thioesterase, which together catalyze six of the seven reactions needed to form DHNA, are virtually the same in roots and leaves (log_2_FD in roots compared to leaves range from −0.22 to 0.29) (Fig. 4). By comparison, in Arabidopsis and maize the log_2_FD of mRNA levels for the same pathway genes in roots compared to leaves range from −6.10 to −1.03, with the exception of DHNA-CoA thioesterase genes, which exhibit log_2_FDs up to 0.98 (Supplementary Figure S3). Similar to its orthologs in Arabidopsis and maize, expression of the black walnut DHNA-CoA synthase gene was found to be lower in roots compared to leaves, although to a lesser degree (log_2_FD of −1.54 in black walnut versus −3.28 and −4.76 in Arabidopsis and maize, respectively) (Fig. 4 and Supplementary Figure S3).

The final three enzymes in the phylloquinone pathway catalyze modifications to the naphthoquinone ring of DHNA that are not present in the structure of juglone. We therefore predicted that unlike genes encoding the enzymes involved in formation of the naphthoquinone ring (reactions 1–5, Fig. 1), the relative root versus leaf expression profiles of genes encoding enzymes that catalyze phylloquinone-specific reactions (reactions 6–8, Fig. 1) in black walnut would follow the same trend as their orthologs in Arabidopsis and maize. Expression of the gene encoding the black walnut DHNA phytyl transferase was found to have a log_2_FD in roots versus leaves of −3.56 (Fig. 4). This value compares with the expression profiles of the Arabidopsis and maize orthologs (log_2_FDs of −4.49 and −10.94, respectively) (Supplementary Figure S3). The log_2_FDs for the black walnut *NDC1* (−2.67) and *demethylphylloquinone methyltransferase* (−3.14) genes were also found to be within the range of the log_2_FDs for their cognate orthologs in Arabidopsis and maize (Supplementary Figure S3). Thus, the RNA-seq results indicate that the relative root versus leaf expression values for all but one of the genes encoding the enzymes forming the naphthoquinone moiety of phylloquinone are higher in black walnut compared to Arabidopsis and maize. At the same time, the relative root versus leaf expression values for black walnut genes encoding enzymes catalyzing phylloquinone-specific reactions are comparable to those of their Arabidopsis and maize orthologs and to other black walnut photosynthesis-related genes revealed by GO enrichment analysis of DEGs (Supplementary Tables S3-6). Similar trends of relative root to leaf gene expression in black walnut were observed by quantitative reverse transcription-PCR using gene-specific primers (Fig. 4). These results support the existence of an alternative fate for DHNA derived from the phylloquinone pathway in black walnut roots.

### Juglone is *de novo* synthesized from an intermediate of the phylloquinone pathway in black walnut roots

**Fig. 5:**
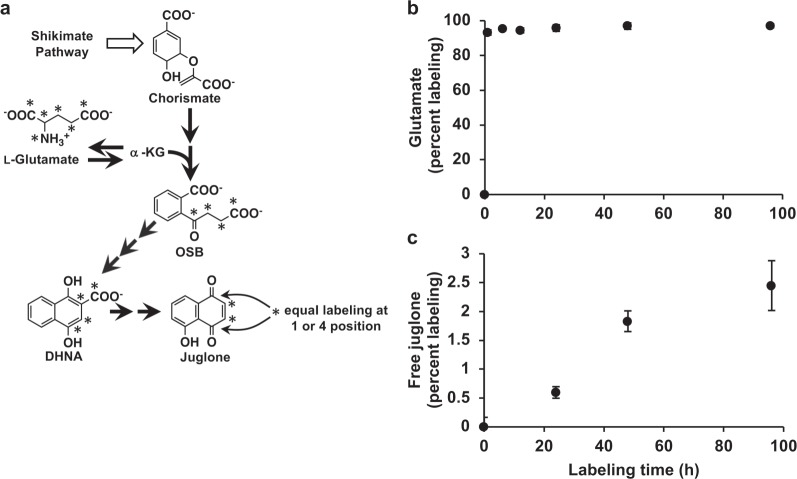
Isotopic labeling of juglone from ^13^C_5_^15^N_1_-l-glutamate (Glu-[^13^C_5_^15^N_1_]). **a** Scheme depicting labeling pattern of Glu-[^13^C_5_^15^N_1_] incorporated into the structure of juglone via DHNA derived from the phylloquinone pathway. **b** Time course of percent labeling of the l-glutamate pool in axenically grown *Juglans nigra* (black walnut) roots fed with 25 mM Glu-[^13^C_5_^15^N_1_]. The glutamate pool was labeled by over 90% within 30 min and remained constant over the 96 h experiment. **c** Time course of percent labeling of the free juglone pool in axenically grown black walnut roots fed with 25 mM Glu-[^13^C_5_^15^N_1_]. Each time point represents the average percent labeling in glutamate or juglone from two independent feeding experiments

Given the relative high expression of phylloquinone pathway genes encoding enzymes involved in synthesizing DHNA in black walnut roots, we next sought to determine if stable isotopically labeled glutamate is incorporated into juglone with the same mass shift as that expected if juglone is derived from an intermediate of the phylloquinone pathway. Because the quinone ring (ring *B*) of phylloquinone originates from glutamate via α-ketoglutarate (Fig. 1)^18^, we therefore assessed the incorporation into juglone of ^13^C_5_^15^N_1_-labeled l-glutamate (Glu-[^13^C_5_^15^N_1_]) fed to axenic black walnut root cultures. We predicted that if the naphthalenoid precursor for juglone is derived from the phylloquinone pathway, then juglone should be labeled by Glu-[^13^C_5_^15^N_1_] with a mass shift of +3 (M3; Fig. 5a).

Young roots were detached from 1-year-old black walnut trees, washed, surface-sterilized, and continuously fed with 25 mM Glu-[^13^C_5_^15^N_1_] for 0, 24, 48, or 96 h in liquid culture. Isotopic abundances of glutamate and free juglone pools were then analyzed by GC-MS. Isotopomers produced from Glu-[^13^C_5_^15^N_1_] fed to roots were distinguished from unlabeled compounds based on mass spectra obtained from GC-MS analyses and natural isotopic abundances were subtracted. After 0.5 h feeding, the glutamate pool in roots was labeled by over 95% and remained constant in root cultures for the duration of the feeding experiment (Fig. 5b). The M3 ^13^C enrichment of the juglone pool from Glu-[^13^C_5_^15^N_1_] increased linearly over 48 h to 1.83% and then began to plateau, reaching 2.45% by 96 h (Fig. 5c). These results therefore demonstrate that juglone can be *de novo* synthesized in roots and provide additional support that the juglone naphthalenoid moiety originates via the phylloquinone pathway.

## Discussion

The ability to synthesize a myriad of 1,4-NQ natural products facilitating plant–plant, plant–microbe, and/or plant–insect interactions has independently evolved across multiple lineages via several different metabolic routes within the flowering plants^8^. Although most pathway genes remain unidentified, biosynthesis of some 1,4-NQ natural products may rely on precursors or intermediates now established to be part of pathways leading to quinones involved in photosynthesis or respiration^8^. Combining comparative transcriptomics with metabolic profiling, we show that phylloquinone pathway genes encoding enzymes involved in forming DHNA are expressed in black walnut roots to support production of a metabolite other than phylloquinone. This result supports the findings of the classical radiotracer study performed by Müller and Leistner^21^, who demonstrated that [1,4-^14^C]-DHNA fed to English walnut leaves could be incorporated into juglone, but without knowledge of phylloquinone pathway enzymes could not postulate the in vivo intermediacy of DHNA. Therefore, to test if DHNA serves as an in planta intermediate in juglone biosynthesis, we fed stable isotopically labeled glutamate to axenic black walnut root cultures and found that Glu-[^13^C_5_^15^N_1_] incorporates into juglone with the same mass shift as that expected for phylloquinone (Fig. 5). Putting together the transcriptomic, metabolomic, and labeling evidence provided here, with the observations of Müller and Leistner^21^, thus indicates that juglone is *de novo* synthesized in black walnut roots from DHNA derived via the phylloquinone pathway. By extension of knowledge about the plant phylloquinone pathway, our work has revealed the complete set of genes/enzymes involved in forming the juglone naphthoquinone ring starting from chorismate.

The black walnut DHNA-CoA synthase gene is expressed three times higher in leaves than roots, but it is still highly expressed in both tissues relative to other phylloquinone pathway genes (Fig. 4). The substrate of DHNA-CoA synthase, OSB-CoA (Fig. 1), has been shown in vitro to be unstable at physiological pH and to spontaneously hydrolyze to its spirodilactone form^35–37^. Recycling of this *a priori* metabolically inactive compound in plants would necessitate the activity of an esterase to convert OSB spirodilactone back to OSB, thereby creating a “futile cycle” within the phylloquinone pathway^18^. It therefore seems reasonable to postulate that in black walnut the DHNA-CoA synthase gene is highly expressed in order to produce enough enzyme to ensure sufficient conversion of OSB-CoA to DHNA-CoA to avoid futile cycling of OSB-CoA back to OSB. Such an expression pattern may have evolved to drive flux toward DHNA to sustain the prolific production of juglone in roots (Fig. 2). In leaves, high DHNA-CoA synthase levels may ensure sufficient carbon allocation to DHNA to support juglone synthesis without depleting the precursor pool available for phylloquinone, which is a vital component of PSI.

In addition to being transported within or between cells, plant natural products are often translocated from the site of synthesis to other tissues or organs^38^. A classic example of this is translocation of the alkaloid nicotine, produced in roots of some *Solanaceous* species, to leaves via the xylem^39^. Many other instances of partitioning synthesis and storage of natural products occur throughout flowering plants suggesting this strategy could be a common feature to protect highly metabolically active cells from cytotoxicity or to better utilize resources by centrally localizing synthesis with subsequent deployment to multiple destinations^40^. However, feeding experiments with Glu-[^13^C_5_^15^N_1_] supplied to isolated black walnut roots performed in this study (Fig. 5), combined with previous tracer studies using English walnut leaves^19–21^, demonstrate that juglone can be *de novo* synthesized in both organs. Thus, while the origin of juglone detected in bark and reproductive organs (Fig. 2) is still unknown, juglone synthesis does not appear to be centrally localized. In addition, the labeling experiment performed in this study demonstrates that *de novo* juglone synthesis can occur in roots without translocation of an immediate precursor from aerial tissues.

**Fig. 6:**
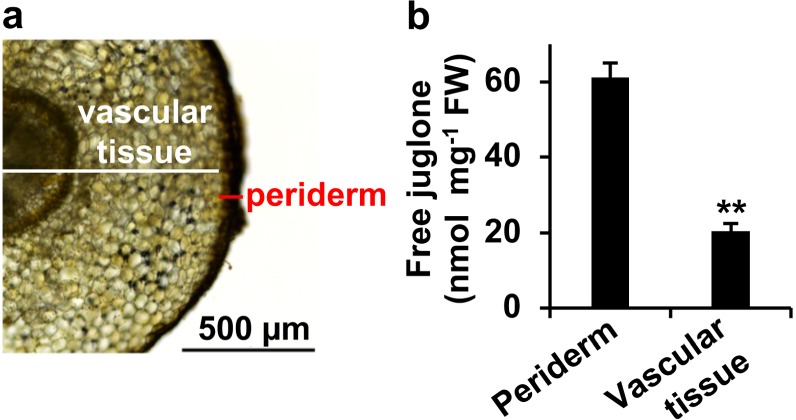
Juglone accumulates in *Juglans nigra* (black walnut) root periderm. **a** Black walnut root cross section depicting dissected sections of periderm and vascular tissue. **b** Profiling of free juglone in periderm and vascular tissue. Data are means ± SEM (*n* = 3 biological replicates), **p *<* 0.001 by Student’s *t*-test relative to periderm

Within black walnut roots, juglone accumulates in the periderm (Fig. 6) from where it is released in large amounts into the rhizosphere to mediate plant–plant and plant–microbe interactions^8^. Because juglone is also present in the root vasculature (Fig. 6), though, it is possible that a portion of the juglone pool in roots is translocated from aerial parts of the plant and/or that juglone pools in some aerial organs originate via translocation from roots. Moreover, it should be investigated if juglone synthesis in roots occurs in the vascular tissue and is transported to the periderm for secretion into the environment. The identification of juglone pathway genes in this study now enables future studies to explore the tissue-level distribution of the juglone pathway in roots.

No herbicide with a novel mode of action has been commercialized in over 25 years^41^. Natural products offer a rich source of new chemical structures and modes of action, but compared to other pesticides and pharmaceuticals, they have been vastly underutilized for developing herbicides^42–44^. Though not yet completely understood, juglone’s herbicidal mode(s) of action appears to be distinct from that of any existing synthetic commercial herbicide^41^, making it a prime candidate for developing a novel natural product-based herbicide. The discovery that the naphthalenoid moiety of juglone originates from DHNA derived from the phylloquinone pathway now raises the prospect of developing metabolic engineering strategies in virtually any tolerant crop species or using green algae, cyanobacteria, or certain menaquinone-synthesizing bacteria as a platform for large-scale juglone production. To achieve such goals, however, the next step is to complete the elucidation of juglone biosynthesis from DHNA. We propose this occurs through DHNA decarboxylation followed by hydroxylation of 1,4-NQ (Fig. 1), the latter of which may be catalyzed by a member(s) of the cytochrome P450 or 2-oxoglutarate/Fe(II)-dependent dioxygenase families^8^. The transcriptomes generated in this study provide candidate lists (Supplementary Tables S10-12) of black walnut genes that may encode the enzymes carrying out the final steps of the juglone pathway. These lists can now be further refined by examining transcriptomes across juglone-producing species and using co-expression analyses with phylloquinone pathway genes to identify target juglone pathway genes for functional testing.

## Disclaimer

Any opinions, findings, and conclusions or recommendations expressed in this material are those of the authors and do not necessarily reflect the views of the National Science Foundation.

## Electronic supplementary material

Supplementary Figures S1-3

Supplementary Tables S1-12

## References

[1] Willis, R. J. *The History of Allelopathy* 1542 (Springer, Netherlands, 2008).

[2] Dana M, Lerner B (2001). Black walnut toxicity. Purdue Univ. Coop. Ext. Serv..

[3] Soderquist CJ (1973). Juglone and allelopathy. J. Chem. Educ..

[4] Massey A (1925). Antagonism of the walnuts (Juglans nigra L. and J Cinera L.) in certain plant associations. Phytopathology.

[5] Babula P (2014). Phytotoxic action of naphthoquinone juglone demonstrated on lettuce seedling roots. Plant Physiol. Biochem..

[6] Chi WC (2011). Identification of transcriptome profiles and signaling pathways for the allelochemical juglone in rice roots. Plant Mol. Biol..

[7] Dayan Franck E., Duke Stephen O. (2009). Biological Activity of Allelochemicals. Plant-derived Natural Products.

[8] Widhalm JR, Rhodes D (2016). Biosynthesis and molecular actions of specialized 1,4-naphthoquinone natural products produced by horticultural plants. Hortic. Res..

[9] van Oostende C, Widhalm JR, Furt F, Ducluzeau AL, Basset GJ (2011). Adv. Bot. Res..

[10] Maeda H, Dudareva N (2012). The shikimate pathway and aromatic amino acid biosynthesis in plants. Annu. Rev. Plant. Biol..

[11] Gross J (2006). A plant locus essential for phylloquinone (vitamin K1) biosynthesis originated from a fusion of four eubacterial genes. J. Biol. Chem..

[12] Garcion C (2008). Characterization and biological function of the ISOCHORISMATE SYNTHASE2 gene of Arabidopsis. Plant Physiol..

[13] Kim HU, Oostende C, Van, Basset GJC, Browse J (2008). The AAE14 gene encodes the Arabidopsis o-succinylbenzoyl-CoA ligase that is essential for phylloquinone synthesis and photosystem-I function. Plant J..

[14] Widhalm JR (2012). Phylloquinone (vitamin K1) biosynthesis in plants: two peroxisomal thioesterases of lactobacillales origin hydrolyze 1,4-dihydroxy-2-naphthoyl-coa. Plant J..

[15] Shimada H (2005). Inactivation and deficiency of core proteins of photosystems I and II caused by genetical phylloquinone and plastoquinone deficiency but retained lamellar structure in a T-DNA mutant of Arabidopsis. Plant J..

[16] Fatihi A (2015). A dedicated type II NADPH dehydrogenase performs the penultimate step in the biosynthesis of bitamin K1 in Synechocystis and Arabidopsis. Plant Cell.

[17] Lohmann A (2006). Deficiency in phylloquinone (vitamin K1) methylation affects prenyl quinone distribution, photosystem I abundance, and anthocyanin accumulation in the Arabidopsis AtmenG mutant. J. Biol. Chem..

[18] Basset GJ, Latimer S, Fatihi A, Soubeyrand E, Block A (2017). Phylloquinone (vitamin K1): occurrence, biosynthesis and functions. Mini Rev. Med. Chem..

[19] Leistner E, Zenk MH (1968). Zur Biogenese von 5-Hydroxy-1.4-naphthochinon (Juglon) in Juglans regia L. Z. Naturforsch. B.

[20] Chung D, Maier UH, Inouye H, Zenk MH (1994). Different mode of incorporation of o-succinylbenzoic acid into the naphthoquinones juglone and lawsone in higher plants. Z. Naturforsch. C.

[21] Müller W, Leistner E (1976). 1,4-Naphthoquinone, an intermediate in juglone (5-hydroxy-1,4-naphthoquinone) biosynthesis. Phytochemistry.

[22] Kolosova N (2004). Isolation of high-quality RNA from gymnosperm and angiosperm trees. Biotechniques.

[23] Martínez-García PJ (2016). The walnut (Juglans regia) genome sequence reveals diversity in genes coding for the biosynthesis of non-structural polyphenols. Plant J..

[24] Grabherr MG (2013). Trinity: reconstructing a full-length transcriptome without a genome from RNA-Seq data. Nat. Biotechnol..

[25] Fu L, Niu B, Zhu Z, Wu S, Li W (2012). CD-HIT: accelerated for clustering the next-generation sequencing data. Bioinformatics.

[26] Li B, Dewey CN (2011). RSEM: accurate transcript quantification from RNA-Seq data with or without a reference genome. BMC Bioinformatics.

[27] Leng N (2013). EBSeq: an empirical Bayes hierarchical model for inference in RNA-seq experiments. Bioinformatics.

[28] Love MI, Huber W, Anders S (2014). Moderated estimation of fold change and dispersion for RNA-seq data with DESeq2. Genome Biol..

[29] Yu G, Wang LG, Han Y, He QY (2012). clusterProfiler: an R Package for comparing biological themes among gene clusters. Omi. A J. Integr. Biol..

[30] Rhodes D, Hogan AL, Deal L, Jamieson GC, Haworth P (1987). Amino acid metabolism of Lemna minor L.: II. Responses to chlorsulfuron. Plant Physiol..

[31] Oostende C, van, Widhalm JR, Basset GJC (2008). Detection and quantification of vitamin K1 quinol in leaf tissues. Phytochemistry.

[32] Bak S (2011). Cytochromes P450. Arabidopsis Book.

[33] Dunand C, Crèvecoeur M, Penel C (2006). Distribution of superoxide and hydrogen peroxide in Arabidopsis root and their influence on root development: possible interaction with peroxidases. New Phytol..

[34] Widhalm JR, Dudareva N (2015). A familiar ring to it: biosynthesis of plant benzoic acids. Mol. Plant.

[35] Dansette P, Azerad R (1970). A new intermediate in naphthoquinone and menaquinone biosynthesis. Biochem. Biophys. Res. Commun..

[36] Heide L, Kolkmann R, Arendt S, Leistner E (1982). Enzymic synthesis of o-succinylbenzoyl-CoA in cell-free extracts of anthraquinone producing Galium mollugo L. cell suspension cultures. Plant Cell Rep..

[37] Meganathan R, Bentley R (1979). Menaquinone (vitamin K2) biosynthesis: conversion of o-succinylbenzoic acid to 1,4-dihydroxy-2-naphthoic acid by Mycobacterium phlei enzymes. J. Bacteriol..

[38] Yazaki K, Sugiyama A, Morita M, Shitan N (2008). Secondary transport as an efficient membrane transport mechanism for plant secondary metabolites. Phytochem. Rev..

[39] Hashimoto T, Yamada Y (2003). New genes in alkaloid metabolism and transport. Curr. Opin. Biotechnol..

[40] Jorgensen ME, Nour-Eldin HH, Halkier BA (2015). Transport of defense compounds from source to sink: Lessons learned from glucosinolates. Trends Plant. Sci..

[41] Dayan FE, Duke SO (2014). Natural compounds as next-generation herbicides. Plant Physiol..

[42] Cantrell CL, Dayan FE, Duke SO (2012). Natural products as sources for new pesticides. J. Nat. Prod..

[43] Adebesin F, Widhalm JR, Lynch JH, McCoy RM, Dudareva N (2018). A peroxisomal thioesterase plays auxiliary roles in plant β-oxidative benzoic acid metabolism. Plant J..

[44] Liu Y (2015). RNA-seq analysis reveals MAPKKK family members related to drought tolerance in maize. PLoS ONE.

