# Supplementary material for: The origin and biosynthesis of the naphthalenoid moiety of juglone in black walnut
Source: Hortic Res. 2018 Nov 1;5:67. doi: 10.1038/s41438-018-0067-5 (PMC6210188; doi:10.1038/s41438-018-0067-5)
Supplement: Supplementary file 1 — Supplementary Figures S1-3 [file 41438_2018_67_MOESM1_ESM.doc]

**SUPPLEMENTARY FIGURE S1**

**Supplementary Figure S1.** Detection of free juglone in plant tissue by HPLC fluorescence. (**a**) On-line excitation scans for juglone and plumbagin with the emission wavelength set at 372 nm. (**b**) On-line emission scans for juglone and plumbagin with the excitation wavelength set at 230 nm. (**c**) HPLC diode array spectrophotometric traces of a juglone standard, a plumbagin standard, and a *Juglans nigra* (black walnut) leaf extract. Juglone elutes at 17.1 minutes and plumbagin elutes at 20.1 minutes. (**d**) HPLC fluorescence traces of the same samples in (c) using the optimized excitation and emission wavelengths determined from the scans presented in (a) and (b).

**SUPPLEMENTARY FIGURE S2**

**Supplementary Figure S2.** Pool sizes of phylloquinone and free juglone in *Juglans nigra* (black walnut) organs used to generate RNA-seq datasets. (**a**) Phylloquinone levels. (**b**) Free juglone levels. Data are means ± SEM (*n* = 3 technical replicates). Different letters indicate significant differences via Analysis of Variance (ANOVA) followed by Post-hoc Tukey test (α = 0.05).

**SUPPLEMENTARY FIGURE S3**

**Supplementary Figure S3.** Relative expression of phylloquinone pathway genes in *Arabidopsis thaliana* and *Zea mays* (maize) roots versus leaves. Unlike *Juglans nigra* (black walnut) roots, all phylloquinone pathway genes involved in synthesizing 1,4-dihydroxynaphthoic acid (DHNA) in Arabidopsis and maize, except those encoding 1,4-dihydroxy-2-naphthoyl-CoA (DHNA-CoA) thioesterases, which are known to be promiscuous enzymes^14,43^, have more than two-fold lower expression in roots. Log_2_ fold-differences (FD) were calculated based on expression of phylloquinone pathway genes in Arabidopsis and maize using publicly available Gene Expression Omnibus datasets. The corresponding accession numbers are GSE87760 (unpublished) and GSE71377^44^. Count data were accessed from control wild-type samples for leaves and roots. AtDHNAT1, *Arabidopsis thaliana* DHNA-CoA thioesterase 1; AtDHNAT2, *Arabidopsis thaliana* DHNA-CoA thioesterase 2. See Figure 1 legend for other abbreviations.
